# Case report of optic atrophy in Dentatorubropallidoluysian Atrophy (DRPLA)

**DOI:** 10.1186/s12883-015-0520-0

**Published:** 2015-12-18

**Authors:** Michael R. Silver, Kapil D. Sethi, Shyamal H. Mehta, Fenwick T. Nichols, John C. Morgan

**Affiliations:** Department of Neurology, Movement Disorders, School of Medicine, Emory University, Atlanta, GA 30322 USA; Department of Neurology, Movement Disorders Program, Medical College of Georgia, Georgia Regents University, 1429 Harper Street, HF-1154, Augusta, GA 30912 USA; Department of Neurology, Movement Disorders, Mayo Clinic, Scottsdale, AZ 85259 USA; Neurology Service, Veterans Affairs Medical Center, One Freedom Way, Augusta, GA 30904 USA; Department of Neurology, Stroke Program, Medical College of Georgia, Georgia Regents University, Augusta, GA 30912 USA

**Keywords:** Dentatorubropallidoluysian Atrophy (DRPLA), Optic atrophy, Ataxia, Dystonia, African American

## Abstract

**Background:**

Dentatorubropallidoluysian atrophy (DRPLA) is a rare autosomal dominant neurodegenerative disease that is associated with numerous movement disorders. Ocular problems also occur with DRPLA with reports of corneal endothelial degeneration in some patients living with the disease. We report a new visual problem associated with DRPLA, optic atrophy.

**Case presentation:**

A 47 year-old man presented complaining of progressive visual loss associated with optic atrophy on ophthalmological evaluation. He gradually developed a progressive ataxia with dystonia. Brain MRI revealed a diffuse leukoencephalopathy. Genetic analysis revealed 62 CAG repeats in one allele of the DRPLA gene and he was diagnosed with DRPLA.

**Conclusion:**

Optic atrophy should be included in the clinical spectrum of DRPLA.

## Background

Dentatorubropallidoluysian Atrophy (DRPLA) is a rare autosomal dominant neurodegenerative disease characterized by a variety of symptoms and signs including progressive ataxia, choreoathetosis, dementia, myoclonus, psychiatric problems, and seizures [1, 2]. DRPLA is most common in Japan and very rare outside of Asia, however there are multiple North American and European pedigrees [2–7]. A large African-American pedigree with “Haw River Syndrome” (HRS) was eventually discovered to have DRPLA [2, 3]. The pathogenesis of DRPLA involves expanded CAG repeats in the DRPLA gene on chromosome 12p13 leading to a polyglutamine expansion in the Atrophin-1 protein and neuronal intranuclear inclusions [8].

DRPLA is associated with visual disturbances in some patients due to corneal endothelial degeneration [9, 10]. To our knowledge, there are no reports of optic atrophy associated with DRPLA. We describe an African-American man with DRPLA who presented with progressive visual loss associated with optic atrophy, gradually progressive ataxia, dystonia and severe leukoencephalopathy.

## Case presentation

**Fig. 1:**
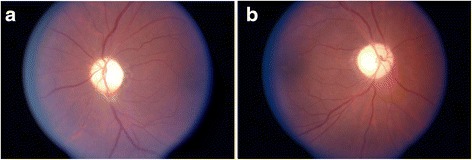
Optic atrophy on fundoscopic exam in DRPLA. Very pale optic discs are evident bilaterally. **a** OS, and (**b**) OD

A 47 year-old African-American man presented to an ophthalmologist for progressive worsening in his vision. He had no prior history of reversible neurological deficits that would be concerning for demyelinating disease and no episodes of sudden visual loss. The temporal field of vision in his left eye was impaired due to an injury he sustained in a football game 25 years earlier, however he noted progressive visual loss bilaterally over the last several years. Examination of his visual acuity with a Snellen chart at the time revealed 20/30 acuity in the right eye and 20/70 acuity in the left eye. Four years earlier, he had 20/30 acuity on the right and 20/50 on the left. Ophthalmological evaluation revealed normal corneas, normal intraocular pressures, optic atrophy bilaterally (Fig. 1), and a swinging flashlight test revealed a left afferent pupillary defect. Given the diagnostic possibilities of a chiasmatic tumor or multiple sclerosis, a brain MRI was ordered and showed diffuse white matter disease involving the pons and the subcortical frontoparietal regions concerning for microischemic small vessel disease. The optic nerves and optic chiasm were also thought to be atrophic by the interpreting neuroradiologist. The patient had no history of exposure to optic neurotoxins such as methanol, ethambutol, or chloroquine. He was diagnosed with hypertension around this time and treated with fosinopril and hydrochlorothiazide. There was no personal or family history of a genetic disorder. The patient’s mother died of diabetes complications at an unknown age and his father died at age 68 of “old age”. There was no known optic atrophy or progressive significant visual loss and no movement disorders in his parents. His brother was known to have “fidgety” movements of his limbs concerning for chorea, but he was never formally diagnosed with any disease. His two children were deceased (one due to a gun-shot wound to the head) and neither child ever displayed any visual problems or neurological problems. He had nine grandchildren and five great grandchildren, none of whom had visual problems or neurological problems.

On initial neurological evaluation he was noted to have dysmetria on both finger-to-nose and heel-to-shin on the left and an unstable tandem gait. A work-up revealed an unremarkable trans-thoracic echocardiogram, elevated total cholesterol at 261, a negative ANA, ESR of 4, a non-reactive RPR, normal vitamin B-12 level, normal blood glucose, normal CBC, negative HIV-1 testing, and a normal serum lactate.

**Fig. 2:**
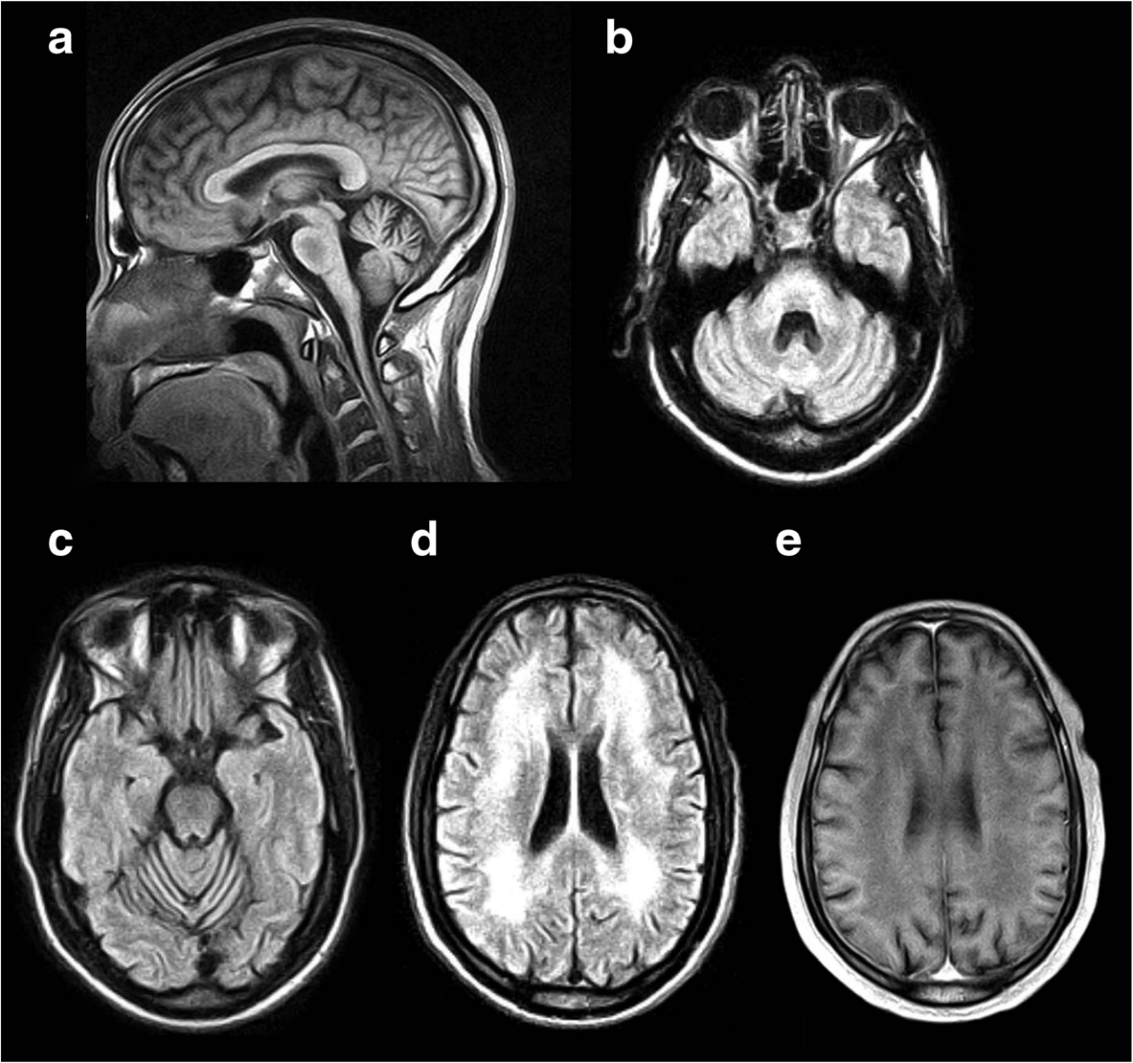
MRI of the brain demonstrating characteristic findings in DRPLA. **a** Mid-sagittal T1-weighted image illustrating cerebellar atrophy and central pontine hypointensity. **b**, **c**, **d** Axial FLAIR images demonstrating atrophy of the dentate with 4^th^ ventricular *ex vacuo* dilatation (**b**), midline cerebellar atrophy (**c**), and confluent hyperintense leukoencephalopathy (**d**). **e** Axial T1-weighted imaging demonstrating confluent white matter hypointensity in approximately the same area as (**d**)

His vision and ataxia progressively worsened and a brain MRI at age 53 revealed severe leukoencephalopathy (Fig. 2). In addition, cerebellar atrophy with fourth ventricle *ex vacuo* dilatation and brain stem volume loss was evident (Fig. 2). Given the progressive nature of his disorder we undertook a search for possible genetic causes of ataxia. Following appropriate counseling, commercially available genetic testing was performed for autosomal dominant causes of ataxia including SCA1, SCA2, SCA3, SCA6, SCA7, SCA8, SCA17, and DRPLA. While negative for all other SCAs, the patient had 62 CAG repeats in one DRPLA allele and 12 CAG repeats in the other allele. He was diagnosed with DRPLA and counseled about the testing results. OPA1 (autosomal dominant optic atrophy) genetic testing was not commercially available and this genetic test was not performed.

At age 57, the patient remained fully oriented, retained 3/3 immediate and remote recall and displayed pseudobulbar affect. He no longer drove due to incoordination and visual problems, but managed his activities of daily living. He had dysarthric, scanning speech, slowed vertical and horizontal saccades, and dystonic posturing of his right upper extremity and face at times. There was no choreoathetosis or myoclonus evident. He had significant dysmetria on finger-to-nose and heel-to-shin bilaterally, dysdiadochokinesia and significant gait ataxia requiring a cane for ambulation.

His uncorrected visual acuity had declined to being able to only count fingers from one foot inward OS and 20/300 OD. Visual fields were severely constricted OS. Slit lamp examination revealed corneas with 2-3+ guttata and endothelial haze bilaterally most likely representing endothelial degeneration due to DRPLA. Intraocular pressures remained normal bilaterally and he had mild bilateral cataracts. Fundoscopy again showed bilateral pale optic discs consistent with optic atrophy, more pronounced in the left eye.

Eleven years after initial presentation the patient was admitted to a nursing home due to inability to care for himself secondary to DRPLA. The patient eventually died after 14 years of disease.

## Conclusions

In this case, we described an African-American man who presented with optic atrophy, ataxia and leukoencephalopathy. Progressive decline in his vision, coordination and gait as well as progression in his leukoencephalopathy was evident upon serial examinations and repeat brain MRI six years later. He was eventually diagnosed with DRPLA following genetic testing. We could not identify other causes of optic atrophy in this patient, and we feel it is most likely related to DRPLA given his extensive white matter disease. While hypertension may have contributed to the progression of his leukoencephalopathy, DRPLA itself is associated with severe leukoencephalopathy over time and was the likely major contributor in our patient [3–5, 7, 11–13].

Unlike the African-American family identified as having HRS that was later discovered to be DRPLA, our patient did not have extensive basal ganglia calcifications, but did have extensive leukoencephalopathy as seen in HRS [3, 4]. Another distinguishing clinical feature of our patient is the absence of significant dementia at present. DRPLA, like SCA3 and Huntington’s disease, can have a quite variable phenotype depending on the number of CAG repeats, the race of the patient and associated comorbidities [1–8, 14].

To our knowledge, DRPLA has not been implicated as a cause of optic atrophy. Typical MRI findings in DRPLA include cortical and cerebellar atrophy and degeneration of the dentatorubral and pallidoluysian systems [11, 12]. Neuropathological findings in DRPLA vary and can include basal ganglia calcifications and central demyelination as well as neuronal loss and astrogliosis in the globus pallidus, dentate nucleus and subthalamic nucleus [1–8, 13]. We cannot identify prior MRI or neuropathological reports of optic atrophy even in older DRPLA patients with significant visual loss and severe leukoencephalopathy [7]. Other than the novel finding of optic atrophy, our patient’s clinical and neuroimaging features are typical of the DRPLA spectrum.

Corneal endothelial degeneration certainly could have contributed to our patient’s progressive visual loss and it is probably much more common in DRPLA than realized. Despite the first description of DRPLA by Smith et al. in 1958 [15], the first report of corneal endothelial degeneration in DRPLA was not until 1999 [9]. The corneal endothelium is derived from neuroectoderm and there is expression of the DRPLA gene in these cells leading to degeneration over time [9, 10]. Corneal endothelial degeneration was present in our patient as evidenced by the endothelial haze and 2–3+ guttata noted on his last ophthalmological examination. The lack of prior reports of optic atrophy associated with DRPLA may be analogous to the long delay in the discovery of corneal endothelial degeneration in these patients. Alternatively, unknown racial, genetic or environmental influences in the setting of a predisposing condition for white matter disease (DRPLA) could have led to the optic atrophy.

The optic nerve and retina are CNS structures and the astrocytes and oligodendrocytes of the optic nerve would theoretically be predisposed to the same DRPLA disease process as in the cerebral subcortical white matter. Atrophin-1 may have a possible role in co-repressing orphan nuclear receptor TLX, involved in retinal progenitor cell proliferation [16]. Other SCAs can be associated with optic atrophy (especially in SCA1) and thinning of the retinal nerve fiber layer by optical coherence tomography (OCT) [17–19]. Interestingly, ocular manifestations are not typically seen in SCA3 mouse models.

It appears that the diffuse leukoencephalopathy associated with DRPLA is more common in patients with a greater number of CAG repeats and is more common in older DRPLA sufferers [7, 8, 13]. White matter degeneration in DRPLA could be due to neurodegeneration from expression of abnormal Atrophin-1 or due to influences on cerebral small vessels leading to diffuse ischemia [7, 8, 13]. Recent research indicates that abnormal metabolism of polysaccharides in endothelial cells, astrocytes and oligodendrocytes and/or microvasculature dysfunction may contribute to white matter damage in DRPLA [13]. Both of these processes are possible contributors to the pathogenesis of optic atrophy we observed in our patient.

Clinicians should be aware that DRPLA can be associated with optic atrophy.

## Consent

Written informed consent was obtained from the patient for publication of this case report and any accompanying images. A copy of the written consent is available for review by the Editor of this journal. Dr. Morgan has also received ethical approval from the IRB for retrospective chart review and case reports regarding patients in his practice.

## Abbreviations

ANA: antinuclear antibody
CBC: complete blood count
DRPLA: Dentatorubropallidoluysian Atrophy
ESR: erythrocyte sedimentation rate
HIV: human immunodeficiency virus
HRS: Haw River Syndrome
MRI: magnetic resonance imaging
OD: right eye
OS: left eye’
RPR: Spinocerebellar Ataxia
